# Evolution of a Novel Muscle Design in Sea Urchins (Echinodermata: Echinoidea)

**DOI:** 10.1371/journal.pone.0037520

**Published:** 2012-05-18

**Authors:** Alexander Ziegler, Leif Schröder, Malte Ogurreck, Cornelius Faber, Thomas Stach

**Affiliations:** 1 Museum of Comparative Zoology, Harvard University, Cambridge, Massachusetts, United States of America; 2 Molecular Imaging Group, Leibniz-Institut für Molekulare Pharmakologie, Berlin, Germany; 3 Institut für Werkstoffforschung, Helmholtz-Zentrum Geesthacht, Geesthacht, Germany; 4 Institut für Klinische Radiologie, Universitätsklinikum Münster, Münster, Germany; 5 Institut für Biologie, Freie Universität Berlin, Berlin, Germany; Columbia University, United States of America

## Abstract

The sea urchin (Echinodermata: Echinoidea) masticatory apparatus, or Aristotle's lantern, is a complex structure composed of numerous hard and soft components. The lantern is powered by various paired and unpaired muscle groups. We describe how one set of these muscles, the lantern protractor muscles, has evolved a specialized morphology. This morphology is characterized by the formation of adaxially-facing lobes perpendicular to the main orientation of the muscle, giving the protractor a frilled aspect in horizontal section. Histological and ultrastructural analyses show that the microstructure of frilled muscles is largely identical to that of conventional, flat muscles. Measurements of muscle dimensions in equally-sized specimens demonstrate that the frilled muscle design, in comparison to that of the flat muscle type, considerably increases muscle volume as well as the muscle's surface directed towards the interradial cavity, a compartment of the peripharyngeal coelom. Scanning electron microscopical observations reveal that the insertions of frilled and flat protractor muscles result in characteristic muscle scars on the stereom, reflecting the shapes of individual muscles. Our comparative study of 49 derived “regular” echinoid species using magnetic resonance imaging (MRI) shows that frilled protractor muscles are found only in taxa belonging to the families Toxopneustidae, Echinometridae, and Strongylocentrotidae. The onset of lobe formation during ontogenesis varies between species of these three families. Because frilled protractor muscles are best observed *in situ*, the application of a non-invasive imaging technique was crucial for the unequivocal identification of this morphological character on a large scale. Although it is currently possible only to speculate on the functional advantages which the frilled muscle morphology might confer, our study forms the anatomical and evolutionary framework for future analyses of this unusual muscle design among sea urchins.

## Introduction

Most extant sea urchins (Echinodermata: Echinoidea) possess a complex masticatory apparatus, the so-called Aristotle's lantern. The lantern is employed by “regular” echinoids (the “Regularia” do not form a monophyletic taxon, hence the quotes) as a gripping apparatus to scrape off encrusting organisms and to feed on larger food items. In sand dollars (Echinoidea: Clypeasteroida), the lantern serves as a crushing device that grinds ingested sediment into finer material. The complex design of sea urchin lanterns has fascinated morphologists throughout the centuries, resulting in a wide array of literature dealing with lantern morphology [1]–[10], lantern physiology [11]–[15], and lantern biomechanics [16]–[20]. According to these studies, the lantern is composed of 40 skeletal elements (i.e., five teeth, five rotulae, ten hemi-pyramids, ten epiphyses, and ten compass elements) as well as numerous soft tissue structures, among them a large number of unpaired and paired muscle groups. One of the latter, the lantern protractor muscles, are the focus of this study. However, we shall first provide the relevant background information for the present contribution by summarizing the current knowledge on lantern muscle morphology.

**Figure 1:**
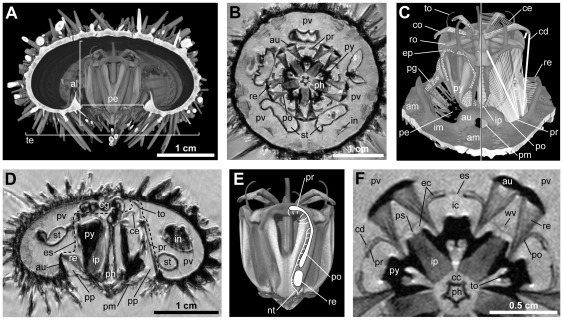
Gross morphology of Aristotle's lantern and corresponding muscles in *Paracentrotus lividus*. (A) Virtual vertical section through a volume-rendered 3D model based on a μCT dataset with 27 µm isotropic voxel resolution. (B) Virtual horizontal section through a MRI dataset with 81 µm isotropic voxel resolution at the level of the retractor muscles. (C) Semi-schematic illustration of the main lantern muscles as well as the compass depressors (right-hand side) and their corresponding insertion sites on skeletal elements (left-hand side). Not to scale. (D) Virtual vertical section through a MRI dataset with 81 µm isotropic voxel resolution at the level of the central oral-aboral axis. The dotted line indicates the exterior septum that separates the peripharyngeal coelom from the perivisceral coelom. (E) Innervation of the protractor, postural, and retractor muscles. Adapted to *P. lividus* from results acquired by Boltt & Ewer [45] and Cobb & Laverack [33] on two closely related species, *Parechinus angulosus* and *Echinus esculentus*. Not to scale. (F) Virtual vertical section through a MRI dataset with a resolution of 50×50×200 µm at the level of the retractor muscles. In horizontal section, the protractor muscles appear as flat bands. al = Aristotle's lantern, am = ambulacrum, au = auricle, cc = central cavity, cd = compass depressor, ce = compass elevator muscle, co = compass, ec = exterior cavity, eg = esophagus, ep = epiphysis, es = exterior septum, ic = interradial cavity, im = interambulacrum, in = intestine, ip = interpyramidal muscle, nt = nerve trunk, pe = peristome, pg = perignathic girdle, ph = pharynx, pm = peristomial membrane, po = postural muscle, pp = peripharyngeal coelom, pr = protractor muscle, ps = perradial septum, pv = perivisceral coelom, py = pyramid, re = retractor muscle, ro = rotula, st = stomach, te = test, to = tooth, wv = water vessel.

In *Paracentrotus lividus* (Lamarck, 1816), a “regular” sea urchin species and one of the few model organisms for studies on Aristotle's lantern (Fig. 1), the masticatory organ is located at the center of the calcareous test, above and within the peristome as well as surrounding the pharynx (Fig. 1A). A horizontal section through the center of the lantern reveals its pentamerous symmetry (Fig. 1B). Although the lantern is predominantly a masticatory device, several of its components do not directly serve in feeding. For example, the compass elevator muscles and the compass depressors (Fig. 1C) aid primarily in respiration by raising and lowering the compass elements [19], [21], [22], while the dental promoter muscles serve to advance the teeth along the pyramids [23], [24]. The compass depressors have been shown to contain primarily mutable collagenous tissue and only a thin muscular layer [25]–[27]. Furthermore, the pharyngeal levator and depressor muscles assist in the formation of food pellets inside the pharynx in most “regular” sea urchins [28], [29]. A set of five tiny, unpaired interepiphyseal muscles is present as well [29], [30].

The four remaining muscle groups are those that are used mainly in mastication. These are the interpyramidal, retractor, protractor, as well as the postural muscles (Fig. 1C). The five interpyramidal muscles are located in-between the pyramids and are used to draw the teeth together [29], [31], [32]. The ten retractor muscles attach to the base of the pyramids as well as to the auricles, adapically-oriented protrusions of the perignathic girdle that surrounds the peristome. The retractors serve in withdrawing the lantern and in pulling the teeth apart [8], [16], [18], [19], [29]. The ten protractor muscles in turn extend from the paired epiphyses to the medial interambulacral parts of the perignathic girdle. Upon contraction, the protractors protrude the lantern, but also move the teeth together. Finally, the ten postural muscles attach to the frontolateral areas of the pyramids as well as to the lateral interambulacral parts of the perignathic girdle. The posturals are in continuation with the protractors [29], but presumably serve to “stabilize the jaws in a particular position in the cycle of opening and closing movements” [33].

Although the lantern is, like the digestive or ambulacral systems, surrounded by a large coelomic space, the perivisceral coelom, it is in fact separated from the latter by a thin membrane, the exterior septum (Fig. 1D). This structure is composed of an epithelial bilayer and separates lantern ossicles and muscles from the perivisceral coelom by enclosing them in the peripharyngeal coelom [34]. An outward movement of the lantern is restricted by the peristomial membrane [35]–[38], another element of the lantern that is partly composed of the mutable collagenous tissue so characteristic of echinoderms [26].

All lantern muscles are derivatives of the different epithelial sheets that constitute the lining of the peripharyngeal coelom. Like most echinoderm muscles, the lantern musculature is composed of smooth muscle fibers [15]. Previous histological and ultrastructural investigations of lantern muscles [8], [10], [29], [31], [33], [39], [40], [41] have revealed that the muscles are contractile structures composed of numerous fascicles. A fascicle is composed of several muscle fibers, each of which represents a single myocyte. The muscle fibers contain innumerous myofilaments of variable thickness that cause the muscle to contract or expand. There are several parallels to the smooth muscle of vertebrates: like these, the echinoderm smooth musculature is made up of fusiform cells [42], and acetylcholine acts as muscle contractant, while nitric oxide and neuropeptides serve as muscle relaxants [43]. Stauber [29] as well as Dolmatov and colleagues [41] have shown that the compass elevator, retractor, protractor, and postural muscles constitute true subepithelial muscles derived from a stratified myoepithelium. The individual muscle fascicles are formed by clusters of ciliated myoepithelial cells that are lined by a basal lamina and that sink into the underlying connective tissue during ontogenesis. The loose connective tissue separates the fascicles from each other and the epithelia [30].

Many muscle fibers are running along the whole length of the muscle, but the muscles are innervated only from the end that faces the lantern [44]. However, the innervations of the retractor, protractor, and postural muscles are independent only to some degree. While the retractor and postural muscles are jointly innervated through the nerve trunk that runs around the base and on the abaxial side of the pyramid (Fig. 1E), the protractor muscle is innervated by a separate nerve trunk that passes along the adaxial side of the pyramid. Both nerve trunks arise from one of the ten hyponeural ganglia, paired structures that lie on either side of the five ambulacral radial nerve cords. These ganglia are abutting the circumoral nerve ring [33], [45]–[47]. However, results obtained for *Eucidaris tribuloides* (Lamarck, 1816) indicate that the nerve trunk running along the adaxial side of the pyramid could also play a role in the innervation of the postural muscle by sending off branches that pierce the pyramid and reach the posturals [48]. Although most authors do not recognize the protractor and postural muscles as separate entities [8], [10], [29], the innervation scheme described above could indeed result in functionally independent muscles and therefore justify a differentiation, a position that we take here.

A closer look at the lantern in horizontal section (Fig. 1F) furthermore reveals that the peripharyngeal coelom is subdivided into various compartments [14], [29]. While the interpyramidal muscles are primarily in contact with the central cavity, the retractors, protractors, and posturals interact predominantly with the interradial cavity. The abaxial side of the protractor muscles, however, is in contact with the exterior cavity, a closed-off coelomic space of the peripharyngeal coelom. Last but not least, the magnetic resonance imaging (MRI) scan of *P. lividus* (Fig. 1F) also shows that in this species the shape of the protractor muscles in horizontal section can best be described as “flat muscle bands” [29]. This observation, however, is in stark contrast to the situation encountered in a related, derived “regular” species, *Echinometra mathaei* (Blainville, 1825), where the protractor muscles exhibit a different shape [49] and have recently been described as “frilled protractor muscles” [50]. Triggered by these findings, it is the aim of the present contribution to better understand this significant divergence in gross morphology of protractor musculature among sea urchins. Using a combination of non-invasive and invasive techniques, we describe the histology and ultrastructure of the frilled protractor muscle encountered in *E. mathaei*, compare these results with those derived from other species, provide measurements of muscle size in representative echinoid taxa, analyze the taxonomic distribution of frilled protractor muscles among derived “regular” sea urchins, and discuss potential functions of this unusual muscle design.

## Results

**Figure 2:**
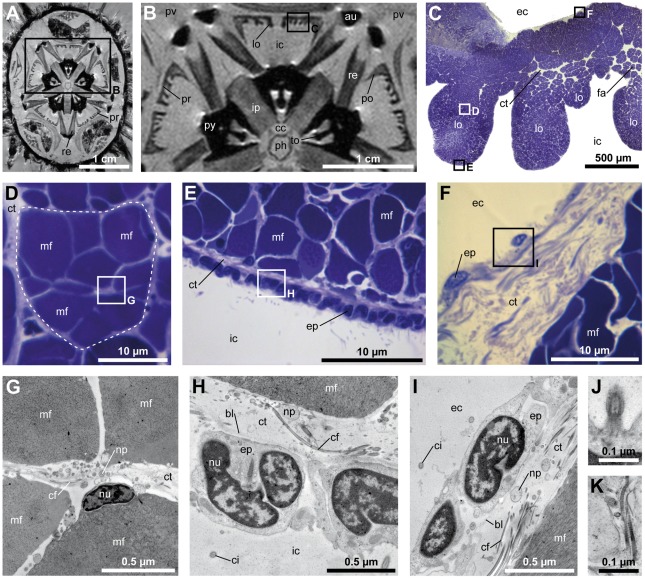
Gross morphology, histology, and ultrastructure of the frilled protractor muscle found in *Echinometra mathaei*. (A) Virtual horizontal section through a MRI dataset with 81 µm isotropic voxel resolution at the level of the retractor muscles. (B) Close-up view. In horizontal section, the protractor muscles appear as frilled bands. (C) Semi-thin section through a frilled protractor muscle. (D) Semi-thin section of a fascicle (indicated by the dotted line). (E) Semi-thin section of the protractor muscle epithelium directed towards the interradial cavity. (F) Semi-thin section of the protractor muscle epithelium directed towards the exterior cavity. (G) Ultra-thin section through the bordering area of four muscle fibers. (H) Ultra-thin section of the ciliated cuboidal epithelium directed towards the interradial cavity. (I) Ultra-thin section of the ciliated epithelium directed towards the exterior cavity. (J) Ultra-thin vertical section through a cilium. All epithelia found covering the protractor muscles are ciliated. (K) Ultra-thin vertical section through a collagen fibril. The presence of collagen fibrils varies between the adaxial and the abaxial connective tissue layers. au = auricle, bl = basal lamina, cc = central cavity, cf = collagen fibril, ci = cilium, ct = connective tissue, ec = exterior cavity, ep = epithelial cell, fa = fascicle, ic = interradial cavity, ip = interpyramidal muscle, lo = lobe, mf = muscle fiber, np = nerve process, nu = nucleus, ph = pharynx, po = postural muscle, pr = protractor muscle, pv = perivisceral coelom, py = pyramid, re = retractor muscle, to = tooth.

The protractor muscles of *Paracentrotus lividus* form “flat bands” in horizontal section (Fig. 1F), whereas the protractor muscles of *Echinometra mathaei* resemble “frilled bands” in horizontal section (Fig. 2).

### Gross morphology of the frilled protractor muscle

The frilled protractor muscle in *E. mathaei* is characterized by the presence of adaxially-facing lobes that extend perpendicular to the general oral-aboral orientation of the muscle. The number of lobes per individual muscle varies from four to seven and the lobes on average attain a width similar to the thickness of the main oral-aboral muscle element (Fig. 2A, B). The adaxial-abaxial length of the lobes of a single protractor muscle varies, being largest at the muscle's interambulacral end and decreasing in length towards the postural muscle. The lobes are continuous and extend from the perignathic girdle to the epiphysis. In *E. mathaei*, the lobes are present only on the adaxial side of the protractors, while they are entirely absent from the retractor, postural, and compass elevator muscles. The lobes are immersed in the coelomic fluid of the interradial cavity.

### Histological and ultrastructural properties of frilled protractor muscles

A semi-thin section of a frilled protractor muscle of *E. mathaei* reveals that the fascicles are, on average, evenly distributed throughout the muscle, although they are slightly more concentrated towards the muscle's adaxial side and within the lobes than they are in the central part of the muscle (Fig. 2C). The fascicles are contained within the connective tissue layer between the two epithelia, and the fascicles found at the center of the muscle are composed of about a dozen muscle fibers of varying cross-sectional shapes and with diameters ranging from about 2–10 µm (Fig. 2D). The individual muscle fibers within a fascicle are separated by a connective tissue matrix with interspersed collagen fibrils and nerve processes. The nuclei of the myocytes are randomly distributed along the axis of the muscle fibers (Fig. 2G). Within a fascicle, the muscle fibers are in contact with each other by *zonulae adherentes*. The adaxial epithelium (i.e., that facing the interradial cavity) is cuboidal and consists of numerous tightly packed ciliated cells (Fig. 2E). The connective tissue layer between the epithelial cells and the underlying muscle fibers is relatively thin and few collagen fibrils can be found within the connective tissue (Fig. 2H). In contrast, the abaxial epithelium (i.e., that facing the exterior cavity) is supported by a comparatively thick connective tissue layer (Fig. 2F) with numerous interspersed collagen fibrils (Fig. 2I). This epithelium consists of varying, mostly squamous epithelial cells, which are loosely scattered on top of the connective tissue layer. The thickness of this layer may vary considerably, depending on its location along the muscle (Fig. 2F, I). The muscle fibers directly underlying the adaxial epithelium are smaller in diameter (Fig. 2E) and are not as tightly packed into fascicles as the ones found at the center of the muscle (Fig. 2D). The basiepithelial nerve plexus in both epithelia is poorly developed. All epithelial cells are ciliated (Fig. 2J) and collagen fibrils (Fig. 2K) can be found interspersed throughout the entire connective tissue layer in varying densities. A comparison of histological data on sea urchin protractor muscles reveals a similar microstructure throughout the entire taxon (Table 1).

**Table 1:** Histological observations on juvenile and adult sea urchin protractor muscles.

Species	Protractor shape	Number of lobes	Fiber diameter	Fascicle distribution	Source
*Eucidaris tribuloides* (Cidaridae), juvenile	Flat	-	1–8 µm	Even	Present study
*Stylocidaris affinis* (Cidaridae), adult	Flat	-	2–6 µm	Uneven, most fascicles on adaxial side	[10]
*Echinocyamus pusillus* (Fibulariidae), adult	Flat	-	2–7 µm	Even	Present study
*Arbacia lixula* (Arbaciidae), adult	Flat	-	2–10 µm	Uneven, most fascicles on adaxial side	[40]
*Echinus esculentus* (Echinidae), adult	Flat	-	2–12 µm	Even, slightly more fascicles on adaxial side	[2], [33], [82], present study
*Paracentrotus lividus* (Parechinidae), adult	Flat	-	2–10 µm	Even, slightly more fascicles on adaxial side	[8], [29], [40], present study
*Psammechinus miliaris* (Parechinidae), adult	Flat	-	2–10 µm	Even, slightly more fascicles on adaxial side	Present study
*Lytechinus variegatus* (Toxopneustidae), adult	Frilled	4–5	3–8 µm	Even, slightly more fascicles on adaxial side	Present study
*Sphaerechinus granularis* (Toxopneustidae), adult	Frilled	4–7	2–10 µm	Even, slightly more fascicles on adaxial side	[40], [57], present study
*Echinometra lucunter* (Echinometridae), juvenile and adult	Frilled	4–6	1.5–10 µm	Even, slightly more fascicles on adaxial side	[39], present study
*Echinometra mathaei* (Echinometridae), adult	Frilled	4–7	2–11 µm	Even, slightly more fascicles on adaxial side	Present study
*Echinometra viridis* (Echinometridae), juvenile	Frilled	4–5	3–8 µm	Even, slightly more fascicles on adaxial side	Present study
*Mesocentrotus nudus* (Strongylocentrotidae), juvenile and adult	Frilled	>3	2–7 µm	Uneven, most fascicles on adaxial side	[41]
*Strongylocentrotus droebachiensis* (Strongylocentrotidae), adult	Frilled	4–5	2–10 µm	Even, slightly more fascicles on adaxial side	Present study
*Strongylocentrotus purpuratus* (Strongylocentrotidae), adult	Frilled	4–6	1–11 µm	Even, slightly more fascicles on adaxial side	Present study

The microstructure of this muscle is largely comparable in sea urchins. Data accumulated from various sources.

### Measurements of protractor muscle dimensions

Measurements of protractor muscles found in small and large specimens from six representative species (Table 2) show that the presence of the lobes leads to an increase in muscle volume as well as to an enlargement of the area of the surface directed towards the interradial cavity. This surface is characterized by a cuboidal epithelium (Fig. 2E, H) that plays an important role in providing the muscle with metabolites [29]. A direct comparison between *P. lividus* (flat) and *E. mathaei* (frilled) shows that specimens of roughly equal test and lantern dimensions exhibit considerable differences in protractor muscle parameters, both in small and large adult specimens (Table 2). In case of these two species, the frilled protractor muscle design provides up to three times more muscle volume than the flat muscle design and about four to five times more surface area directed towards the interradial cavity. However, if averaged out across the six representative species for which data are available both for small and large specimens (Table 2), the volume of the frilled muscle is found to be approximately doubled, while the surface area directed towards the interradial cavity increases by a factor of about three to four. These measurements take into account that muscle fiber density within the protractor muscle is largely comparable throughout derived “regular” sea urchins (Table 1) and that the slightly conical oral-aboral shape of the protractor muscle is normalized by using only values derived from measurements at the mid-level of the muscle.

**Table 2:** Measurements of test, lantern, and protractor muscle dimensions in different representative sea urchin species.

	*Arbacia lixula* (small)	*Arbacia lixula* (large)	*Echinus esculentus* (small)	*Echinus esculentus* (large)	*Paracentrotus lividus* (small)	*Paracentrotus lividus* (large)	*Sphaerechinus granularis* (small)	*Sphaerechinus granularis* (large)	*Echinometra mathaei* (small)	*Echinometra mathaei* (large)	*Strongylocentrotuspurpuratus* (small)	*Strongylocentrotuspurpuratus* (large)
Muscle shape	Flat		Flat		Flat		Frilled		Frilled		Frilled	
Test diameter [cm]	2.40	5.10	2.60	8.00	2.50	5.50	2.60	8.00	2.50	5.50	1.90	5.00
Test height [cm]	1.10	2.00	1.40	5.20	1.10	2.20	1.50	5.10	1.50	3.10	1.10	2.60
Lantern diameter [cm]	0.90	1.70	0.90	2.00	0.90	1.50	1.00	2.40	1.20	1.70	0.70	1.50
Lantern height [cm]	0.90	1.70	1.00	2.10	1.00	1.60	1.10	2.50	1.20	1.70	0.70	1.50
Protractor length [mm]	7.0	13.00	7.00	12.00	6.30	12.00	7.10	17.00	8.50	15.00	4.50	10.10
Protractor width [mm]	1.60	3.50	1.30	3.60	1.40	2.30	2.50	4.60	1.90	3.10	1.40	3.10
Protractor thickness [mm]	0.25	0.26	0.26	0.54	0.18	0.23	0.14	0.40	0.16	0.18	0.18	0.30
Lobe length [mm]	-	-	-	-	-	-	0.31	0.55	0.18	0.53	-	0.35
Lobe width [mm]	-	-	-	-	-	-	0.28	0.36	0.19	0.38	-	0.27
Protractor area [mm^2^]	0.40	0.91	0.39	1.94	0.25	0.53	0.78	2.83	0.47	1.57	0.25	1.40
**Protractor volume [mm^3^]**	**2.80**	**11.83**	**2.73**	**23.28**	**1.58**	**6.36**	**5.54**	**48.11**	**4.00**	**23.55**	**1.13**	**14.14**
**Protractor surface area [mm^2^]**	**11.20**	**45.50**	**9.10**	**43.20**	**5.04**	**27.60**	**39.76**	**171.70**	**31.45**	**126.00**	**6.30**	**67.67**

These values are based on 3D MRI scans of a single specimen per species and size group. The muscle parameters were measured at the mid-level of each of the ten protractor muscles and then averaged. In case of the frilled protractor muscles, the calculations of muscle area, volume, and surface area are based on the presence of five lobes on average. The surface area of the protractor muscle is that oriented towards the interradial cavity. The values for protractor muscle volume and surface area (last two lines, in bold) served as the basis for the conclusions presented in Fig. 7.

In order to measure the effect of protractor muscle contraction on muscle thickness and shape, MRI was performed on two species that happened to be fixed with a strongly inclined lantern. For each species, a single specimen was available. The resulting contraction of the protractors led to a thickening of the flat muscle by 39% (*Echinus esculentus* Linnaeus, 1758) and in a thickening of the lobes of the frilled muscle by 23% (*Sphaerechinus granularis* (Lamarck, 1816)). In both specimens, the characteristic shape of the respective protractor muscle, i.e. flat or frilled, was present also after contraction.

### Presence of muscle scars on the stereom

**Figure 3:**
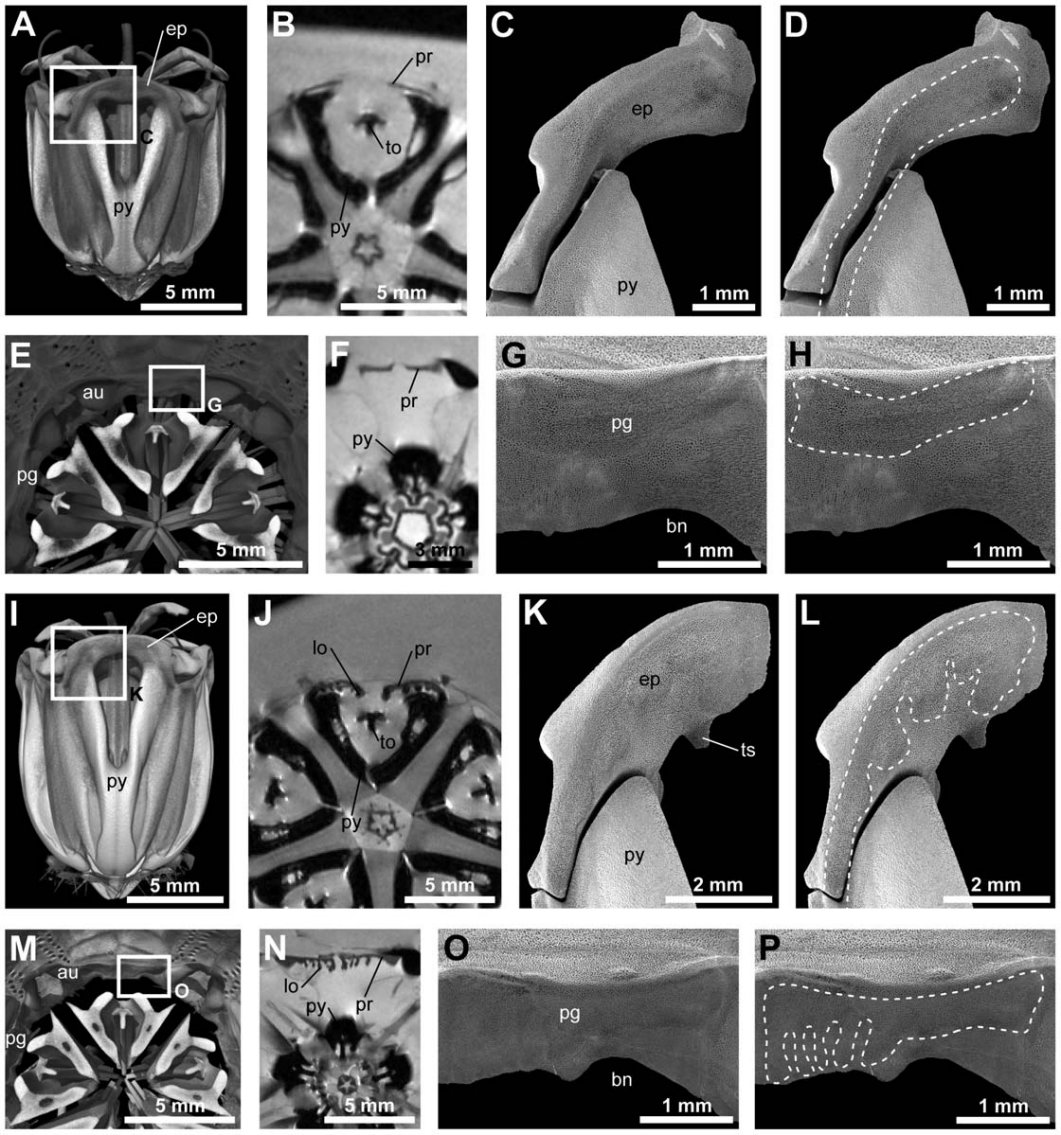
Comparison of muscle scars created by flat (A–H, *Paracentrotus lividus*) and frilled (I–P, *Echinometra mathaei*) protractor muscles on skeletal elements. (A, I) Volume-rendered models of the lantern based on μCT datasets with 27 µm isotropic voxel resolution. The boxes indicate the areas shown in C, D and K, L. (B, J) Virtual horizontal section through MRI datasets with 78×78×500 µm resolution showing the flat and frilled protractor muscles prior to their insertion on the epiphysis. (C, D, K, L) SEM micrographs of the muscle scars created by flat and frilled protractor muscles on the epiphysis and upper pyramid. The dotted lines indicate the outline of the protractor muscle. (K, L, E, M) Volume-rendered models of lantern and perignathic girdle based on μCT datasets with 27 µm isotropic voxel resolution. The boxes indicate the location of the interambulacral insertion site of the protractor muscle on the perignathic girdle. (F, N) Virtual horizontal section through MRI datasets with 78×78×500 µm resolution showing the protractor muscles prior to their insertion on the perignathic girdle. (G, H, O, P) SEM micrograph of the muscle scars created by flat and frilled protractor muscles on the perignathic girdle. The dotted lines indicate the outline of the protractor muscle. au = auricle, bn = buccal notch, ep = epiphysis, lo = lobe, pg = perignathic girdle, pr = protractor muscle, py = pyramid, to = tooth, ts = tooth support.

At the skeletal insertion site of the protractor muscle, the basal lamina of the coelomic epithelium merges into tendon fibrils that interlock with the stereom and simultaneously are attached to the finger-shaped ends of muscle fibers [29]. Such an arrangement should result in differences between flat and frilled protractor muscle scars on the stereom, and this was indeed observed (Fig. 3). The flat protractor muscle of *P. lividus* inserts onto epiphysis (Fig. 3A) and perignathic girdle (Fig. 3E). The muscle retains its flat aspect until its insertion on the stereom at both ends (Fig. 3B, F). The corresponding muscle scars reflect the flat aspect of the entire muscle (Fig. 3C, D, G, H). Likewise, the frilled protractor muscle of *E. mathaei* inserts onto epiphysis (Fig. 3I) and perignathic girdle (Fig. 3M). As the muscle maintains a frilled aspect throughout its entire length (Fig. 3J, N), the muscle scars on epiphysis and perignathic girdle bear the imprint of the main muscle element as well as those of the individual, adaxially-facing lobes (Fig. 3K, L, O, P). Corresponding observations on muscle scars were made in other species analyzed by scanning electron microscopy (SEM): *Arbacia lixula* (Linnaeus, 1758) (flat), *E. esculentus* (flat), *S. granularis* (frilled), and *Strongylocentrotus purpuratus* (Stimpson, 1857) (frilled).

Furthermore, the epiphysis in *E. mathaei* bears a tooth support (Fig. 3K), a skeletal structure not present in *P. lividus* (Fig. 3C). A tooth support is also present in *S. granularis* and *S. purpuratus*, but it is absent in *A. lixula* and *E. esculentus*. Furthermore, the insertion site of the protractor muscle on the perignathic girdle in *E. esculentus* and *P. lividus* is characterized by the flat protractor muscle occupying about half of the available height (Fig. 3H), but in *A. lixula* it occupies the entire height as the perignathic girdle is comparatively narrower in this species. While the frilled part of the muscle occupies the entire height of the perignathic girdle in *E. mathaei* (Fig. 3P), it occupies only about two-thirds of the height in *S. granularis* and *S. purpuratus*.

### Relationship between protractor muscles and the peripharyngeal coelom

**Figure 4:**
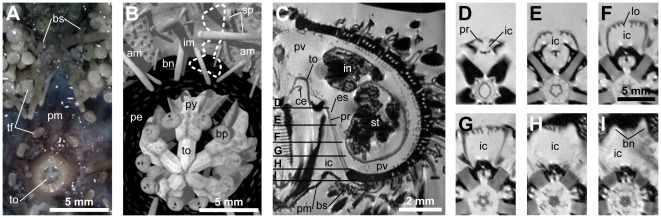
Illustration of the close interrelationship between lantern protractor muscles and buccal sacs in derived “regular” sea urchins, exemplified by *Strongylocentrotus purpuratus*. (A) Photograph of the oral part of the interambulacrum showing the location of the paired buccal sacs. (B) Volume-rendered model of a μCT dataset with 27 µm isotropic voxel resolution showing the same view as in (A), but soft tissues are inapparent due to the type of analysis employed (i.e., X-ray). The dotted line indicates the location of a single buccal sac. (C) Virtual vertical section through a MRI dataset with 42 µm isotropic voxel resolution. The lumen of the buccal sacs is continuous with the interradial cavity. The labels marked (D–I) indicate the location of the horizontal sections shown hereafter. (D–I) Virtual horizontal sections through a MRI dataset with 78×78×500 µm resolution. The protractor muscles are located directly above the buccal notches. am = ambulacrum, bn = buccal notch, bp = buccal plate, bs = buccal sac, ce = compass elevator muscle, es = exterior septum, ic = interradial cavity, im = interambulacrum, in = intestine, lo = lobe, pe = peristome, pm = peristomial membrane, pr = protractor muscle, pv = perivisceral coelom, py = pyramid, sp = spine, st = stomach, tf = tube foot, to = tooth.

Because the lobes of frilled protractor muscles are entirely immersed in coelomic fluid (Fig. 2A–C), the precise location of the protractor muscles and their interplay with the compartments of the peripharyngeal coelom are important in the context of this study. The interradial cavity is indirectly in contact with seawater through the so-called buccal sacs (Fig. 4). These paired, thin-walled, branching pouches are located interambulacrally on the exterior side of the test and are somewhat hidden and protected by neighboring tube feet and spines (Fig. 4A). The location of the buccal sacs can be inferred from the denuded test as well, as the buccal sacs are positioned right below the paired buccal notches of the perignathic girdle (Fig. 4B). The lumen of the buccal sacs is in continuation with the lumen of the interradial cavity (Fig. 4C). The protractor muscles are located above the buccal notches and consequently the lobes of the frilled protractor muscles are situated on top of the opening of the buccal sacs into the interradial cavity (Fig. 4D–I).

### Occurence of frilled protractor muscles across sea urchin taxa

**Figure 5:**
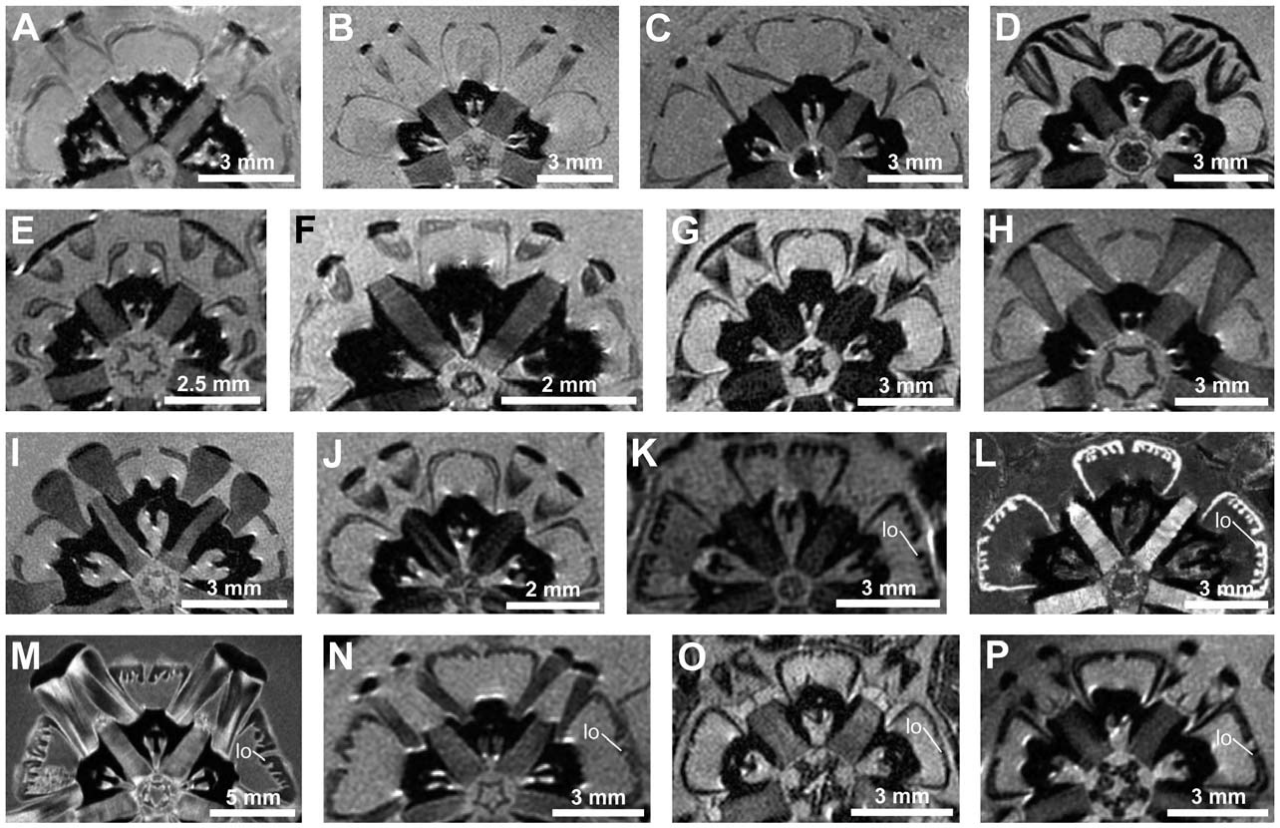
Comparison of protractor muscle shape in selected derived “regular” sea urchin species. Frilled protractor muscles can only be found in sea urchin species of the families Toxopneustidae, Echinometridae, and Strongylocentrotidae (K–P). See Fig. 6 for a phylogeny of the Echinoidea, while Table 3 lists character distribution in all 49 echinacean species analyzed in this study. (A) *Stomopneustes variolaris* (Stomopneustidae). (B) *Arbacia dufresnii* (Arbaciidae). (C) *Parasalenia gratiosa* (Parasaleniidae). (D) *Temnopleurus toreumaticus* and (E) *Pseudechinus magellanicus* (Temnopleuridae). (F) *Trigonocidaris albida* (Trigonocidaridae). (G) *Polyechinus agulhensis* and (H) *Sterechinus neumayeri* (Echinidae). (I) *Parechinus angulosus* and (J) *Psammechinus microtuberculatus* (Parechinidae). (K) *Toxopneustes pileolus* and (L) *Sphaerechinus granularis* (Toxopneustidae). (M) *Echinometra lucunter* and (N) *Heterocentrotus mammilatus* (Echinometridae). (O) *Pseudocentrotus depressus* and (P) *Hemicentrotus pulcherrimus* (Strongylocentrotidae). (A–E), (G–K), and (N–P) based on MRI datasets with 50×50×200 µm resolution. (F) based on a MRI dataset with 32 µm isotropic voxel resolution. (L, M) based on MRI datasets with 78×78×500 µm resolution. lo = lobe.

**Figure 6:**
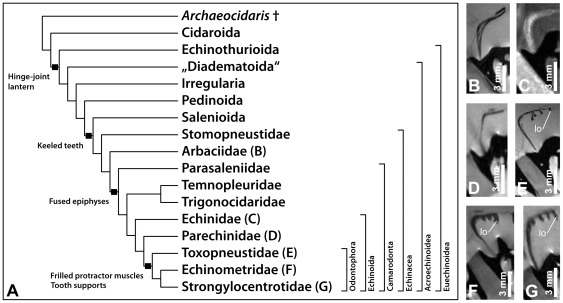
The distribution of frilled protractor muscles is in support of the taxon Odontophora. (A) Phylogeny of sea urchins (Echinodermata: Echinoidea) based on results obtained by Kroh & Smith [67]. The four major events of putative improvements in lantern mechanics have been mapped onto the tree. (B–G) Virtual horizontal sections through MRI datasets with 78×78×500 µm resolution of the lanterns of large adult sea urchins with about 5–8 cm test diameter. The species analyzed represent six families: (B) *Arbacia lixula* (Arbaciidae), (C) *Echinus esculentus* (Echinidae), (D) *Paracentrotus lividus* (Parechinidae), (E) *Sphaerechinus granularis* (Toxopneustidae), (F) *Echinometra mathaei* (Echinometridae), and (G) *Strongylocentrotus purpuratus* (Strongylocentrotidae). lo = lobe.

The distribution of frilled protractor muscles was analyzed by studying 49 echinacean species (Echinoidea: Echinacea). Frilled protractor muscles can only be found in taxa belonging to the families Toxopneustidae, Echinometridae, and Strongylocentrotidae (Fig. 5, Table 3). However, no lobes could be identified on the protractor muscles of one toxopneustid species (*Gymnechinus robillardi* (de Loriol, 1883)) and one echinometrid species (*Caenocentrotus gibbosus* (L. Agassiz in L. Agassiz & Desor, 1846). When adult specimens of about 1–3.5 cm test diameter are analyzed, the relative size of the lobes varies considerably among the species in the three families. In this size group (of adult sea urchins), the lobes are most prominent in toxopneustids and echinometrids (Fig. 5K–N), while strongylocentrotids only weakly display the character state (Fig. 5O, P). However, the relative size of the lobes changes during sea urchin growth (Table 2) and large adult specimens clearly exhibit them (Figs. 4D–I, 6E–G). In contrast, the lobes remain entirely absent in large specimens of species that do not belong to the Toxopneustidae, Echinometridae, or Strongylocentrotidae (Fig. 6B–D). Analysis of the previously [51]–[54] acquired 3D MRI datasets of representative species from all non-echinacean sea urchin taxa that possess a lantern (i.e., Cidaroida, Echinothurioida, “Diadematoida”, Pedinoida, Salenioida, and Clypeasteroida) reveals that frilled protractor muscles are absent from all of these animals.

**Table 3:** Distribution of frilled protractor muscles in derived “regular” sea urchins.

Taxon	Protractor shape	Test diameter	Dataset resolution	Specimen ID
**Stomopneustidae Mortensen, 1903**				
*Stomopneustes variolaris* (Lamarck, 1816)	Flat	2.08 cm	(81 µm)^3^	USNM E45930
**Arbaciidae Gray, 1855**				
*Arbacia dufresnii* (Blainville, 1825)	Flat	2.34 cm	50×50×200 µm	ZMB Ech 2222
*Arbacia lixula* (Linnaeus, 1758)	Flat	2.40 cm	(44 µm)^3^	BMNH 1952.3.26.31–36
*Arbaciella* (Mortensen, 1910)	n/a	-	-	-
*Coelopleurus* sp.	Flat	2.16 cm	(60 µm)^3^	ZMB Ech 7412
*Dialithocidaris* A. Agassiz, 1898	n/a	-	-	-
*Habrocidaris* A. Agassiz & H.L. Clark, 1907	n/a	-	-	-
*Podocidaris* sp.	Flat	0.90 cm	(31 µm)^3^	ZMB Ech 7409
*Pygmaeocidaris* Döderlein, 1905	n/a	-	-	-
*Sexpyga* Shigei, 1975	n/a	-	-	-
*Tetrapygus niger* (Molina, 1782)	Flat	1.57 cm	50×50×200 µm	ZMB Ech 1346
**Temnopleuridae A. Agassiz, 1872**				
*Amblypneustes pallidus* (Lamarck, 1816)	Flat	2.17 cm	50×50×200 µm	ZMB Ech 6334
*Erbechinus* Jeannet, 1935	n/a	-	-	-
*Holopneustes inflatus* (A. Agassiz, 1872)	Flat	2.35 cm	50×50×200 µm	ZMB Ech 2639
*Mespilia globulus* (Linnaeus, 1758)	Flat	1.95 cm	(44 µm)^3^	ZMB Ech 5620
*Microcyphus* (L. Agassiz, in L. Agassiz & Desor, 1846)	n/a	-	-	-
*Opechinus* Desor, 1856	n/a	-	-	-
*Paratrema* Koehler, 1927	n/a	-	-	-
*Printechinus* Koehler, 1927	n/a	-	-	-
*Pseudechinus magellanicus* (Philippi, 1857)	Flat	2.13 cm	50×50×200 µm	ZMB Ech 2188
*Salmaciella* Mortensen, 1942	n/a	-	-	-
*Salmacis sphaeroides* (Linnaeus, 1758)	Flat	1.63 cm	50×50×200 µm	ZMB Ech 4337
*Temnopleurus michaelseni* (Döderlein, 1914)	Flat	1.40 cm	50×50×200 µm	ZMB Ech 6331
*Temnopleurus reevesii* (Gray, 1855)	Flat	1.77 cm	50×50×200 µm	ZMB Ech 3588
*Temnopleurus toreumaticus* (Leske, 1778)	Flat	2.35 cm	50×50×200 µm	ZMB Ech 2802
*Temnotrema* A. Agassiz, 1864	n/a	-	-	-
**Trigonocidaridae Mortensen, 1903**				
*Asterechinus* Mortensen, 1942	n/a	-	-	-
*Desmechinus* H.L. Clark, 1923	n/a	-	-	-
*Genocidaris maculata* A. Agassiz, 1869	Flat	0.84 cm	(36 µm)^3^	ZMB Ech 5827
*Hypsiechinus* Mortensen, 1903	n/a	-	-	-
*Prionechinus* A. Agassiz, 1879	n/a	-	-	-
*Trigonocidaris albida* A. Agassiz, 1869	Flat	1.46 cm	(32 µm)^3^	ZSM 20012468
**Parasaleniidae Mortensen, 1903**				
*Parasalenia gratiosa* A. Agassiz, 1863	Flat	2.58 cm	(79 µm)^3^	BMNH 1983.2.15.7
**Echinidae Gray, 1825**				
*Dermechinus* Mortensen, 1942	n/a	-	-	-
*Echinus esculentus* Linnaeus, 1758	Flat	2.60 cm	(81 µm)^3^	ZMB Ech 4340
*Gracilechinus acutus* (Lamarck, 1816)	Flat	1.63 cm	50×50×200 µm	ZMB Ech 3714
*Gracilechinus alexandri* (Danielssen & Koren, 1883)	Flat	1.88 cm	50×50×200 µm	ZMB Ech 4340
*Polyechinus agulhensis* (Döderlein, 1905)	Flat	2.26 cm	50×50×200 µm	ZMB Ech 7219
*Sterechinus agassizii* Mortensen, 1910	Flat	1.78 cm	50×50×200 µm	BMNH 1914.8.12.126–127
*Sterechinus antarcticus* Koehler, 1901	Flat	2.28 cm	50×50×200 µm	ZMB Ech 5439
*Sterechinus neumayeri* (Meissner, 1900)	Flat	2.50 cm	50×50×200 µm	ZMB Ech 5442
**Parechinidae Mortensen, 1903**				
*Loxechinus albus* (Molina, 1782)	Flat	2.67 cm	50×50×200 µm	BMNH 1966.5.1.61–75
*Paracentrotus lividus* (Lamarck, 1816)	Flat	2.50 cm	(81 µm)^3^	ZMB Ech 7406
*Parechinus angulosus* (Leske, 1778)	Flat	3.12 cm	50×50×200 µm	ZMB Ech 5644
*Psammechinus microtuberculatus* (Blainville, 1825)	Flat	2.20 cm	50×50×200 µm	ZMB Ech 4770
*Psammechinus miliaris* (P.L.S. Müller, 1771)	Flat	2.35 cm	(44 µm)^3^	Private collection
**Toxopneustidae Troschel, 1872**				
*Goniopneustes* Duncan, 1889	n/a	-	-	-
*Gymnechinus robillardi* (de Loriol, 1883)	Flat	2.19 cm	50×50×200 µm	BMNH 1890.6.27.5–8
*Lytechinus variegatus* (Lamarck, 1816)	Frilled	1.91 cm	50×50×200 µm	ZMB Ech 5517
*Nudechinus scotiopremnus* H.L. Clark, 1912	Frilled	2.30 cm	50×50×200 µm	ZMB Ech 6130
*Pseudoboletia* Troschel, 1869	n/a	-	-	-
*Sphaerechinus granularis* (Lamarck, 1816)	Frilled	2.60 cm	(81 µm)^3^	ZMB Ech 2366
*Toxopneustes pileolus* (Lamarck, 1816)	Frilled	1.50 cm	50×50×200 µm	ZMB Ech 3871
*Tripneustes gratilla* (Linnaeus, 1758)	Frilled	3.13 cm	78×78×500 µm	ZMB Ech 1527
*Tripneustes ventricosus* (Lamarck, 1816)	Frilled	2.36 cm	50×50×200 µm	ZMB Ech 5498
**Echinometridae Gray, 1825**				
*Caenocentrotus gibbosus* (L. Agassiz, in L. Agassiz & Desor, 1846)	Flat	2.50 cm	50×50×200 µm	ZMB Ech 5405
*Colobocentrotus* Brandt, 1835	n/a	-	-	-
*Echinometra lucunter* (Linnaeus, 1758)	Frilled	3.40 cm	78×78×500 µm	ZMK Mortensen collection
*Echinometra mathaei* (Blainville, 1825)	Frilled	2.50 cm	(81 µm)^3^	BMNH 1969.5.1.61–75
*Echinometra viridis* A. Agassiz, 1863	Frilled	2.14 cm	50×50×200 µm	ZMB Ech 5503
*Echinostrephus molaris* (Blainville, 1825)	Frilled	1.58 cm	50×50×200 µm	ZMB Ech 4000
*Evechinus* Verrill, 1871	n/a	-	-	-
*Heliocidaris crassispina* (A. Agassiz, 1863)	Frilled	1.99 cm	50×50×200 µm	ZMB Ech 6424
*Heliocidaris erythrogramma* (Valenciennes, 1846)	Frilled	2.19 cm	50×50×200 µm	ZMB Ech 5745
*Heterocentrotus mammilatus* (Linnaeus, 1758)	Frilled	1.65 cm	50×50×200 µm	ZMB Ech 1567
*Podophora atrata* (Linnaeus, 1758)	Frilled	2.52 cm	50×50×200 µm	ZMB Ech 4985
*Selenechinus* de Meijere, 1904	n/a	-	-	-
*Zenocentrotus* A.H. Clark, 1932	n/a	-	-	-
**Strongylocentrotidae Gregory, 1900**				
*Hemicentrotus pulcherrimus* (A. Agassiz, 1863)	Frilled	2.65 cm	50×50×200 µm	ZMB Ech 6425
*Mesocentrotus nudus* (A. Agassiz, 1863)	Frilled	-	-	Dolmatov et al. 2007
*Pseudocentrotus depressus* (A. Agassiz, 1863)	Frilled	2.56 cm	50×50×200 µm	ZMB Ech 6426
*Strongylocentrotus droebachiensis* (O.F. Müller, 1776)	Frilled	2.26 cm	50×50×200 µm	ZMB Ech 4422
*Strongylocentrotus purpuratus* (Stimpson, 1857)	Frilled	1.90 cm	(42 µm)^3^	CASIZ 5724

A total of 49 echinacean (Echinoidea: Echinacea) species were analyzed by MRI, but this table also lists those genera for which no data are yet available. Representative species from all other sea urchin taxa that possess a lantern, and which have previously been analyzed by MRI (i.e., Cidaroida, Echinothurioida, “Diadematoida”, Pedinoida, Salenioida, Clypeasteroida), do not have frilled protractor muscles. See [54] for a full list of sea urchin species analyzed using MRI. Taxonomic arrangement of species according to Kroh & Mooi [83]. n/a = not available.

## Discussion

S. Lovén [5] was the first author to depict frilled protractor muscles in his drawings of lantern gross morphology in *Echinometra lucunter* (Linnaeus, 1758). But like other authors who later provided similar images of lantern anatomy in taxa with frilled muscles [6], [55], [56], Lovén did not make specific reference to this unique muscle design. While some of the more detailed studies on sea urchin protractor muscle histology or ultrastructure in species with frilled muscles also did not mention this peculiar morphology [40], [57], publications by Lavallard and colleagues [39] as well as Dolmatov and colleagues [41] did provide precise descriptions of the frilled protractor muscle and its lobes, both in juvenile and adult specimens of *E. lucunter* and *Mesocentrotus nudus* (A. Agassiz, 1863). However, these two studies did not include any further species, and hence, the restriction of the frilled protractor muscle design to certain taxa of derived “regular” sea urchins remained unnoticed.

As the observation of the complex lantern gross morphology should ideally be performed *in situ* in order to avoid dissection artifacts, we employed MRI, a non-invasive imaging technique specifically suited for studies on soft tissues of invertebrate as well as vertebrate organisms [51], [58]. Although this approach has been discussed controversially [59], it must be regarded as crucial for the unequivocal identification of the frilled muscle design present in a restricted set of sea urchin taxa. Our study provides the first description of the distribution of frilled protractor muscles across derived “regular” sea urchin taxa. The unprecedented, broad taxon sampling presented here permits to identify those species that should serve as model organisms for future functional and physiological studies involving Aristotle's lantern.

### Morphology, histology, and ultrastructure of frilled protractor muscles

The gross morphology of the frilled protractor muscle does not vary significantly between different species. The lobes are always situated on the adaxial side of the muscle. All taxa with frilled protractor muscles that were analyzed possess four or more lobes per individual muscle (Table 1), although the number of lobes often was found to vary between the ten protractor muscles located within a single specimen. The frilled aspect is clearly not an artifact resulting from muscle contraction, as the muscle scars of the lobes are visible on the stereom (Fig. 3K, L, O, P), and flat protractors that were observed in *Echinus esculentus* remained entirely flat even after contraction. It is also not the case that the lobes will inevitably occur in sea urchin species once they have reached a certain size, as lobes could not be identified in specimens not belonging to the three families with frilled muscles, even when the animals had attained a relatively large test diameter (Fig. 6B–D, Table 2).

Here, we have taken the position that the protractor muscle is functionally different from the adjoining postural muscle. This view is primarily based on the observation that the innervation of the two muscles has different origins (Fig. 1E), although it must be mentioned that we were not able to identify the nerves originating from the adaxial nerve trunk and piercing the pyramid that had been described for *Eucidaris tribuloides* [48]. A more detailed histological approach would be necessary to pinpoint the likely presence of these nerves in derived “regular” sea urchins. The evolutionary origin of lantern muscle innervation is certainly of a more general interest because postural and protractor muscles are derived from the retractor muscle, and the protractor in turn constitutes an adapical extension of the postural muscle [10].

Although we experienced considerable difficulties with conventional histological analyses using museum material, the sections nonetheless permitted us to identify the presence or absence of lobes and to measure other parameters (Table 1). The process of lobe formation has not yet been fully described and based on our data we are not able to provide further clues. However, our analyses reveal that the lobes may occur at different time points during ontogenesis (Toxopneustidae and Echinometridae as opposed to Strongylocentrotidae, see Table 2). The lobes first appear as small bumps, but continue to grow as the animal ages [39], [41]. We can only hypothesize that either an increase in the rate of muscle fiber production along the bumps in the epithelial layer, an inhibition of muscle fiber production in the interjacent furrows, or a combination of both leads to the growth of the lobes. A more detailed histological and ultrastructural study involving selected growth stages could provide valuable insights into this process.

**Figure 7:**
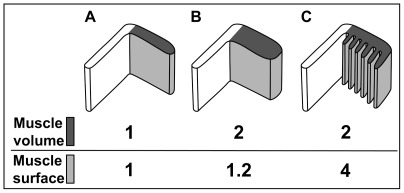
Schematic illustration of general differences in lantern protractor muscle morphology and resulting changes in the relation of muscle volume to muscle surface. An increase in volume of the flat and thin protractor muscle (A) can either result in a flat and thick (B) or a frilled and thin (C) muscle design. However, only the frilled protractor muscle design considerably increases muscle volume (dark grey) as well as the muscle surface directed towards the interradial cavity (light grey). These values are based on measurements provided in Table 2.

Our measurements reveal that the frilled protractor muscle design permits not only an increase in the number of muscle fibers (expressed in muscle volume), but also an increase in the surface area that is directed towards the interradial cavity (Table 2). Furthermore, the analyses show that two major pathways may have contributed to increase protractor muscle strength in “regular” sea urchins (Fig. 7). Derived from a thin protractor muscle as observed in *Arbacia lixula* and *Paracentrotus lividus* (Fig. 7A), the protractor muscle is either thickened as in *E. esculentus* (Fig. 7B) or lobes are formed as in *Sphaerechinus granularis*, *E. mathaei*, and *Strongylocentrotus purpuratus* (Fig. 7C). Both approaches result in an increase of muscle volume, but only the frilled muscle design leads to an additional increase in the surface area directed towards the interradial cavity. The measurements provided here must be treated with some caution, however, as they are based on values derived from a single specimen per species and size group. Higher specimen numbers would certainly be necessary to provide a more robust statistical framework for comparative studies on protractor muscle performance in species with flat and frilled muscles.

Interestingly, another muscle of the lantern is also characterized by considerable folding of its surface area, i.e., the interpyramidal muscle [29], [31], [32]. However, the characteristic folding of this muscle is present in all sea urchin taxa that possess a lantern [10], and this morphology is likely to be an adaptation to the continuous growth of the skeletal element onto which this muscle is inserted, i.e. the pyramid [32]. Furthermore, histological data suggest considerable folding of the adaxial side of the compass depressors in *P. lividus* [8], [21], although this folding does not seem to be present throughout the depressors' entire length [29].

The effect of changes in the echinoid stereom structure at muscle insertion sites has been described in detail [60], [61]. Our observations reveal that flat and frilled protractor muscles result in conspicuous muscle scars on the stereom, thereby reflecting the individual muscle's shape. These muscle scars are not always readily visible, especially when the lobes of the frilled protractor muscle are located close to each other upon insertion. However, the presence of muscle scars at insertion sites on both ends of the muscle implies that the individual lobes are continuous and that the fascicles they contain can exert mechanical force on both the epiphysis and the perignathic girdle.

Finally, our combined data show that the microstructure of protractor muscles throughout the taxon is quite homogenous. Although the distribution of fascicles within the connective tissue matrix was found to vary (Table 1), it is at present not possible to attribute any phylogenetic significance to this observation. Such deviations might also have been caused by differences in fixation protocols, level and plane of sectioning, or state of preservation of the material. Our results for *E. mathaei* are, with regard to most of the histological and ultrastructural data obtained (Fig. 2), in line with the observations on the protractor muscle in *E. lucunter* [39] and *M. nudus* [41]. Like these authors, we observed slight differences in fascicle density within the muscle as well as significant differences in the composition of the adaxial and abaxial epithelia covering the muscle. Because the fascicles are formed through myoepithelial cells sinking into the underlying connective tissue [29], [41], the fascicles directly underneath the epithelia are not expected to be fully developed, in contrast to those encountered at the center of the muscle. Of particular interest is the stark difference between the abaxial and adaxial epithelia, and our results confirm similar observations on flat as well as frilled species [29], [39]–[41]. The cuboidal, tightly packed adaxial epithelial cells with their underlying thin connective tissue layer are suggestive of a strong capacity for nutrient, gas, and waste transport. In contrast, the mostly squamous, loosely scattered abaxial epithelial cells with their underlying thick connective tissue layer are more reminiscent of a diffusion barrier.

### Function of frilled protractor muscles

As the function of this unusual muscle type found in selected sea urchin taxa remains unclear, we here discuss several hypotheses on the possible benefits which frilled protractor muscles could confer.

Our attempt to correlate the presence of frilled muscles with data on sea urchin size and shape failed, as we were unable to find any parameter, including peristome size or shape, test diameter or height, lantern size or shape, or test eccentricity that would permit us to infer the type of protractor muscle. Because the lantern is used predominantly in mastication, frilled muscles might constitute an adaptation to certain types of food or feeding. The data available at present [62] suggest that the taxa included in this study are all opportunistic omnivores with herbivorous habits, making it impossible to pinpoint a specific adaptation to particular food items or modes of feeding. In addition, the presence of frilled protractors does not correlate with specific sea urchin behaviors that are related to the presence of a lantern like, for example, boring or biting. Such behaviors are exhibited only by a few selected taxa mostly within, but also outside of the Odontophora Kroh & Smith, 2010 (Fig. 6A), a recently erected taxon comprising the three families with frilled protractor muscles. However, in particular the echinometrids, a clade with species that possess prominent frilled protractor muscles, are involved in boring and biting [63], [64], while most toxopneustids and strongylocentrotids are not. Unfortunately, sea urchin habitats equally do not provide any clue as to the function of the frilled protractor muscle. Species with frilled muscles can be found both in tropical and cold waters, from the intertidal zone down to the deep sea, precluding a correlation of the presence of frilled protractors with bathymetric range, geographical distribution, water temperature, substrate, or salinity.

However, a closer look at lantern hard parts reveals that a skeletal protrusion of the epiphysis, the tooth support (Fig. 3K), does indeed correlate with the presence of frilled protractor muscles. The tooth support has so far been described only in toxopneustids, echinometrids, and strongylocentrotids [65]–[67]. Its precise function remains unexplored, but the presence of this skeletal element could result in altered lantern mechanics by pushing the tooth away from the epiphysis. In turn, the presence of this element could necessitate a stronger protractor muscle, or vice versa, a strengthened protractor could necessitate a tooth support. However, the development of a stronger protractor muscle has been observed in non-odontophoran taxa as well, but is accomplished here by the simple thickening of the protractor muscle (Fig. 7B) instead of the development of lobes (Fig. 7C). A detailed comparative biomechanical study involving some of the representative species mentioned in the present contribution could shed light on a potential improvement of lantern mechanics in derived “regular” sea urchin taxa. Such an improvement would constitute the latest in a series of evolutionary changes of lantern performance (Fig. 6A), which include the evolution of the hinge-joint lantern in the Euechinoidea, the presence of keeled teeth in derived “regular” echinoids, and the fusion of the epiphyses in the Camarodonta [67], [68]. Apart from a function connected to the presence of skeletal elements, the specific morphology of frilled protractor muscles could also provide a stabilizing function as their lobes might prevent lateral shear of the main protractor muscle element. In addition, contraction and relaxation of the lobes could lead to a more rapid exchange of the coelomic fluid located at the fluid-tissue boundary along the muscle's surface. By squeezing out the fluid located in-between the lobes, such action would prevent the formation of a nutrient- and oxygen-depleted as well as waste product-saturated layer of fluid close to the muscle.

Instead of serving a mechanical purpose, the presence of frilled protractor muscles with their considerable increase in muscle surface area (Fig. 7C) could also be related to an improvement of the overall metabolism of this muscle tissue, because coelomic fluid acts as the main agent for nutrient, gas, and waste transport in sea urchins [12], [69]. This argumentation is in line with the fact that the water vascular system (i.e., tube feet, ampullae, ring canal, radial canals) is the primary means of external gas exchange in sea urchins [70]. In addition to this system, the coelomic fluid contained within the perivisceral coelom has also been assumed to play a role in bringing nutrients, oxygen, or waste products to and from the various tissues [69]. Furthermore, the displacement of perivisceral fluids is partly mediated through protrusion and retraction of the lantern [71]. For example, contraction of the compass elevator muscles raises the compasses which then stretch the exterior septum, drawing fluid from the buccal sacs into the interradial cavity. The location of the protractor muscles right above the buccal sacs is therefore very likely to be of importance in particular for oxygen supply. The latter has been shown to be accomplished for the Aristotle's lantern through the peripharyngeal coelom and not the perivisceral coelom [13]. Although the precise function of the buccal sacs still remains largely unexplored [72], previous studies have already suggested that, apart from their role in excretion, buccal sacs also serve in supplying the peripharyngeal coelom with oxygen [12], [73]. Despite the fact that the combined respiratory surface of all ten buccal sacs constitutes only about 1% of the combined respiratory surface of all tube feet [74], the experimental removal of buccal sacs leads to a significant reduction of oxygen uptake into the peripharyngeal coelom [70].

As the presence of a “well-developed circulatory system” appears to constitute a prerequisite for the emergence of the “sophisticated muscular system of the lantern” [14], the sub-division of the peripharyngeal coelom into central cavity, interradial cavity, exterior cavity, and other pouches is indicative of an improved efficiency in nutrient and gas transport to and from the various lantern muscles. For example, folding of the surface area directed towards the interradial cavity as observed in frilled protractor muscles could lead to an improved oxygenation of muscle fibers located in the deeper parts of the muscle, which could result in a better overall muscle performance. However, the lantern retractor muscles of species with frilled protractor muscles are compact, thick muscles that are clearly not frilled, but are nevertheless able to perform. A detailed physiological study of protractor musculature and its interaction with the different compartments of the peripharyngeal coelom will be necessary to obtain a better understanding of metabolic processes. In addition to the MRI protocols used in this study for morphological inferences, NMR-based spectroscopy techniques performed on living specimens could provide helpful insight into sea urchin lantern physiology [58].

### Evolution of frilled protractor muscles

The distributional pattern of the character “frilled protractor muscle” strongly suggests a presence only in those taxa that belong to the families Toxopneustidae, Echinometridae, and Strongylocentrotidae (Table 3). If mapped onto the latest phylogeny of derived “regular” sea urchin taxa (Fig. 6A), the character is likely to have evolved in the stem lineage of the Odontophora. Due to the relative complexity of this morphological character and its overall similarity between different species, we regard a convergent evolution as highly unlikely. However, we were not able to include all echinacean genera into our analysis (Table 3), and two species nested within the Odontophora do possess flat protractor muscles (*Gymnechinus robillardi* and *Caenocentrotus gibbosus*). Whether the absence of lobes in these two species is related to the size of the specimens analyzed or is rather suggestive of a character loss in these species needs to be addressed in future studies based on more mature specimens.

Unfortunately, no phylogenetic analysis encompassing all echinacean genera is currently available. This informational void applies also to every single echinacean family. However, species identification keys provided by T. Mortensen [65], [66] and subsequent phylogenetic studies on selected taxa permit us to tentatively place the two genera *Gymnechinus* and *Caenocentrotus* at a basal position within their respective families (i.e., Toxopneustidae and Echinometridae). If the absence of frilled protractor muscles could indeed be confirmed in these two genera, this would undoubtedly hint at a convergent evolution of frilled protractor muscles in all three families of the Odontophora. Nonetheless, despite the current lack of support from molecular analyses [75]–[78], the simultaneous presence of tooth support and frilled protractor muscles in Toxopneustidae, Echinometridae, and Strongylocentrotidae is in strong support of the taxon Odontophora.

We also investigated whether a similar muscle design had already been observed in other metazoan taxa. It is unknown from vertebrates, and we only found a single invertebrate group with a similar muscle morphology: the horseshoe worms (Lophotrochozoa: Phoronida), a small taxon of stalked marine filter-feeders. These animals exhibit a considerable degree of extension of the muscle surface area in the longitudinal muscles of the trunk. Form and arrangement of these specialized muscles are used as taxonomic characters [79], [80]. The smooth phoronid trunk musculature shares with the echinoid lantern musculature the absence of a vascular system as well as the presence of individual myoepithelial lobes immersed in coelomic liquid. As in sea urchins, coelomic fluid acts as the main medium for nutrient, gas, and waste transport in horseshoe worms [81].

### Conclusions

Our study demonstrates that the application of non-invasive imaging techniques, in particular MRI, permits to integrate large numbers of valuable museum specimens into comparative morphological studies. The broad taxon sampling employed here allows to identify those species that should in future be considered as model organisms for physiological, biomechanical, and morphological studies on sea urchin lanterns with flat and frilled protractor muscles. The data presented here suggest, for the first time, that frilled protractor muscles have evolved only once in sea urchins. These modified muscles provide the lantern system with increased strength as well as an enlarged surface for metabolic exchange with the surrounding coelomic fluid. Their presence correlates with the occurrence of the skeletal tooth support and is therefore in support of the Odontophora hypothesis. Our comparative study of animal musculature reveals that frilled muscles constitute a noteworthy exception among Metazoa. The present data lead us to suggest that the frilled protractor muscle design found in selected derived “regular” sea urchins can be seen as the latest of an evolutionary series of morphological changes that improve the performance of Aristotle's lantern.

## Materials and Methods

### Magnetic resonance imaging (MRI)

Whole fixed adult sea urchins of about 1–3.5 cm test diameter were scanned using a 9.4 T nuclear magnetic resonance (NMR) scanner equipped for imaging (Bruker Biospin GmbH, Germany). Scanning was performed using a RARE 2D protocol with 3×3 cm field of view, 600×600 pixel matrix size, 50×50 µm in-plane resolution, and a slice thickness of 200 µm. Acquisition time per sample varied from about 10–20 min depending on the number of slices used (30–100). Table 3 provides a full list of sea urchin species analyzed in this study. Lanterns of larger sea urchin specimens (5–8 cm test diameter) were dissected out and imaged using a 7 T small animal MRI scanner (Bruker Biospin GmbH, Germany). These species were *Arbacia lixula* (Linnaeus, 1758), *Echinus esculentus* Linnaeus, 1758, *Paracentrotus lividus* (Lamarck, 1816), *Sphaerechinus granularis* (Lamarck, 1816), *Echinometra mathaei* (Blainville, 1825), and *Strongylocentrotus purpuratus* (Stimpson, 1857). Imaging was performed using a RARE 2D protocol with 4×4 cm field of view, 512×512 pixel matrix size, 78×78 µm in-plane resolution, and 500 µm slice thickness. Acquisition times per sample varied between 13–18 min, depending on the number of averages used (3–5). In addition, whole fixed adult sea urchin specimens measuring about 1–3.5 cm in test diameter were imaged using 7 T and 9.4 T small animal MRI scanners and a 17.6 T NMR scanner equipped for imaging (Bruker Biospin GmbH, Germany). The protocols employed were either FLASH 3D or RARE 3D with 31–81 µm isotropic resolution. Scanning was performed during overnight measurements. Ziegler (in press) provides a full list of sea urchin species scanned using MRI. Specimens from the following collections were available for scanning: British Museum of Natural History (BMNH, London, UK), California Academy of Sciences Invertebrate Zoology (CASIZ, San Francisco, CA, USA), United States National Museum (USNM, Washington, DC, USA), Zoologisches Museum Berlin (ZMB, Berlin, Germany), Zoologisk Museum København (ZMK, København, Denmark), Zoologische Staatssammlung München (ZSM, München, Germany). All samples in this study were contrasted using Magnevist (Bayer HealthCare GmbH, Germany) at a final concentration of 2 mM. More detailed MRI protocols have been published by Ziegler & Mueller [50] as well as Ziegler and colleagues [51]. The datasets were analyzed using the ImageJ (NIH, MD, USA) Volume Viewer plugin.

### Micro-computed tomography (μCT)

Scanning was accomplished at the *Helmholtz-Zentrum Geesthacht* outstation at the *Deutsches Elektronen-Synchrotron* using a Phoenix Nanotom X-ray tube tomography system equipped with a tungsten X-ray source (GE Sensing & Inspection Technologies GmbH, Germany). Scanning parameters were 100 kV source voltage, 160 µA source current, 0.2 mm copper filter, 750 ms exposure time, 1440 angular steps over 360° with 2 averaged images per rotation position, 2304×2304 pixel detector size, and about 1 h 20 min scan time. Image reconstruction was accomplished using the software DatosX Reconstruction 1.5 (GE Sensing & Inspection Technologies GmbH, Germany). The original voxel resolution of the datasets was 13.91 µm; however, only compressed datasets (2× binning) with about 27 µm voxel resolution were used in this study. The specimens analyzed were *P. lividus* (ZMB Ech 7406), *E. mathaei* (BMNH 1969.5.1.61–75), and *S. purpuratus* (CASIZ 5724). The datasets were visualized using the software myVGL 2.1 (Volume Graphics GmbH, Germany).

### Histology

The protractor muscles of eleven formalin-fixed, alcohol-preserved specimens were prepared for conventional histological study. The species examined were *Eucidaris tribuloides* (Lamarck, 1816), *Echinocyamus pusillus* (O.F. Müller, 1776), *E. esculentus*, *Psammechinus miliaris* (P.L.S. Müller, 1771), *Lytechinus variegatus* (Lamarck, 1816), *P. lividus*, *S. granularis*, *Echinometra viridis* (A. Agassiz, 1863), *Echinometra lucunter* (Linnaeus, 1758), *Strongylocentrotus droebachiensis* (O.F. Müller, 1776), and *S. purpuratus*. For light microscopy, single protractor muscles of adult specimens were dissected out or, alternatively, entire juvenile specimens were sectioned. The samples were decalcified in 2% nitric acid (in case of the whole juvenile specimens), dehydrated in a graded ethanol series, methylbenzoate, and butanol, and finally embedded in paraplast (Kendall, MA, USA). Thick sections (6 µm) were prepared using a 2050 Supercut microtome (Reichert-Jung GmbH, Germany) with steel blades (Thermo Shandon, MI, USA). Sections were digitally recorded with a BX 51 light microscope equipped with a BX-UCB digital color camera (Olympus, Japan).

### Transmission electron microscopy (TEM)

Living specimens of *E. mathaei* were purchased from an aquarium supply store. Two specimens were relaxed in 7% MgCl_2_ in seawater for 10 min before dissection along the midline of the test. Compasses and the exterior septum were lifted off the lantern and protractor and retractor muscles were then cut out for TEM observation. The tissue was fixed for 20 h at 4°C in 2.5% glutaraldehyde buffered with cacodylate and NaCl at pH 7.2. After washing with cacodylate, postfixation with 1% OsO_4_ for 1 h at 4°C, and washing with cacodylate and MilliQ Aqua bidest. (Millipore Corporation, MA, USA), the tissue was dehydrated for contrasting in a graded ethanol and acetone series, and embedded in Araldite. Semi-thin sectioning (1 µm) was performed on an Ultracut S ultramicrotome (Reichert GmbH, Germany) using a Histo Jumbo diamond knife (Diatome, Switzerland). The sections were stained using Epoxy tissue stain (Science Services, Germany). The slides were examined and photographed with a BX 51 light microscope equipped with a BX-UCB digital color camera (Olympus, Japan). Ultra-thin sectioning (50–70 nm) was performed on an Ultracut S ultramicrotome (Reichert GmbH, Germany) using a Histo Jumbo diamond knife (Diatome, Switzerland). The sections were stained with 2% uranyl acetate and 2% lead citrate and examined using a CM 120 BioTWIN transmission electron microscope (Philips, The Netherlands).

### Scanning electron microscopy (SEM)

Observations of skeletal elements were performed on the same six large sea urchin species as for MRI. After dissection along the midline of the test, a single pyramid as well as part of the perignathic girdle were cut out and immersed in 5% NaOCl for 3 h. The skeletal elements were then washed in MilliQ Aqua bidest. (Millipore Corporation, MA, USA) and air-dried. For SEM, the objects were placed on imaging tables, the epiphysis was glued back on if found disarticulated, and the samples were prepared according to conventional SEM protocols and observed at 15 kV with a Quanta 200 scanning electron microscope (FEI, OR, USA).
